# BMSC-Exosomes Carry Mutant HIF-1α for Improving Angiogenesis and Osteogenesis in Critical-Sized Calvarial Defects

**DOI:** 10.3389/fbioe.2020.565561

**Published:** 2020-11-19

**Authors:** Chenting Ying, Rui Wang, Zhenlin Wang, Jie Tao, Wenjing Yin, Jieyuan Zhang, Chengqing Yi, Xin Qi, Dan Han

**Affiliations:** ^1^Department of Orthopedics, Shanghai General Hospital, Shanghai Jiao Tong University School of Medicine, Shanghai, China; ^2^Department of Critical Care Medicine, Shanghai General Hospital, Shanghai Jiao Tong University School of Medicine, Shanghai, China; ^3^Department of Neurosurgery, Shanghai General Hospital, Shanghai Jiao Tong University School of Medicine, Shanghai, China; ^4^Department of Orthopedics, Shanghai Jiao Tong University Affiliated Sixth People’s Hospital, Shanghai, China; ^5^Department of Emergency Medicine and Intensive Care, Shanghai Songjiang Clinical Medical College of Nanjing Medical University, Shanghai, China

**Keywords:** BMSC-Exos-HIF1α, bone defects, osteogenesis, vascularization, β-TCP scaffolds

## Abstract

Repair and reconstruction of critical-sized bone defects has always been a difficult task in orthopedics. Hypoxia inducible factor-1α (HIF-1α) plays an important role in bone defect repair, it has the dual function of promoting osteogenesis and vascular regeneration, but it is quickly degraded by the body under normoxic conditions. Previously we prepared mutant HIF-1α, which has been shown to efficiently maintain cellular expression under normoxic conditions. In this study, we evaluated for the first time the role of exosomes of rat bone marrow mesenchymal stem cell carry mutant HIF-1α (BMSC-Exos-HIF1α) in repairing critical-sized bone defects. Evaluation of the effects of BMSC-Exos-HIF1α on bone marrow mesenchymal stem cells (BMSCs) proliferation and osteogenic differentiation by cell proliferation assay, alkaline phosphatase activity assay, alizarin red staining, real-time quantitative polymerase chain reaction. BMSC-Exos-HIF1α was loaded onto the β-TCP stent implanted in the bone defect area using a rat cranial critical-sized bone defect model, and new bone formation and neovascularization were detected *in vivo* by micro-CT, fluorescence labeling analysis, Microfil perfusion, histology and immunohistochemical analysis. *In vitro* results showed that BMSC-Exos-HIF1α stimulated the proliferation of BMSCs and up-regulated the expression level of bone-related genes, which was superior to bone marrow MSC exosomes (BMSC-Exos). *In vivo* results showed that BMSC-Exos-HIF1α combined with β-TCP scaffold promoted new bone regeneration and neovascularization in the bone defect area, and the effect was better than that of BMSC-Exos combined with β-TCP scaffold. In this study, the results showed that BMSC-Exos-HIF1α stimulated the proliferation and osteogenic differentiation of BMSCs and that BMSC-Exos-HIF1α combined with β-TCP scaffolds could repair critical-sized bone defects by promoting new bone regeneration and neovascularization.

## Introduction

Repair and reconstruction of critical-sized bone defects caused by trauma, bone tumor, infection, etc. has always been a difficult task in the field of orthopedic treatment (Zhao et al., 2015). Current clinical methods of repairing bone defects include autologous bone grafting, allogeneic bone grafting, and combination of scaffold material with growth factors or cells. Autologous bone grafting is often considered the gold standard for repairing bone defects, but its use has a number of limitations, including a high incidence of donor sites, limited availability, relatively high and unpredictable autologous absorption (Benic and Hämmerle, 2014). Homozygous allogeneic bone grafting limits its potential clinical application due to high susceptibility to immune rejection (Behrend et al., 2016). With the development of bone tissue engineering, the development of scaffold materials has made great progress, beta-tricalcium phosphate (β-TCP) has good bioactivity, biodegradability, biocompatibility and is an ideal material for bone tissue repair and replacement (Gao et al., 2014; Oryan and Alidadi, 2018), but it doesn’t exactly mimic the physiological function of bone tissue.

Hypoxia-inducible factor-1α (HIF-1α) plays an important role in the repair of bone defects (Yellowley and Genetos, 2019). On the one hand, HIF-1α promotes the differentiation of bone marrow mesenchymal stem cells (BMSCs) to osteogenic cells and enhances osteogenic capacity (Costa et al., 2017). On the other hand, HIF-1α mobilizes endothelial progenitor cells (EPCs) through the CXCL12/CXCR4 biotaxis to homing to the ischemic site and promotes microvascular regeneration at the bone defect site (Bianco et al., 2015; Kawakami et al., 2015; Fu et al., 2018; Ju et al., 2018; Zhao et al., 2018). Good blood supply is a prerequisite for bone tissue regeneration (Zhang et al., 2017). Therefore, we hope to repair the critical-sized bone defect by some way to make HIF-1α continuously expressed at the site of the bone defect through the dual role of HIF-1α in promoting osteogenesis and angiogenesis.

However, the physiologically expressed HIF-1α is degraded within minutes under normoxic conditions, and the preparation of active HIF-1α that is not easily degraded under normoxic conditions is a key issue that needs to be addressed. We have previously genetically engineered three amino acid loci of the HIF-1α subunit functional region (CDS region) to mutate to alanine at 402, 564, and 803, and confirmed that mutant HIF-1α can effectively maintain cellular expression under normoxic conditions, further *in vivo* tests have found that mutant HIF-1α modified bone marrow mesenchymal stem cells (BMSC-HIF1α) effectively promote angiogenesis and have not found this graft to cause tumorigenicity in experimental animals (Qi et al., 2013; Yang et al., 2014).

In recent years, studies have shown that the role of stem cells in tissue repair is accomplished through the activation of peripheral receptor cells by exosomes secreted by their paracrine mechanism (Tan et al., 2020; Wang et al., 2020; Zhang B. et al., 2020). The exosome molecule is 50–150 nm in diameter, and it mainly contains cytokines, growth factors and other proteins, lipids, coding, or non-coding RNAs similar to the cells of origin, and participates in cell communication, cell migration, vascular regeneration and other processes, which plays an important role in the regulation of cell physiological functions (Li et al., 2018). Application of exosomes in heterozygous animals does not induce a significant immune response (Roccaro et al., 2013). The application of BMSC-exosomes carrying mutant HIF1α (BMSC-Exos-HIF1α) to repair critical-sized bone defects has not yet been reported.

The purpose of this study is to evaluate the role of BMSC-Exos-HIF1α in repairing critical-sized bone defects. Our results show for the first time that BMSC-Exos-HIF1α can effectively stimulate the proliferation and osteogenic differentiation of BMSCs, and the effect is better than that of BMSC-Exos; BMSC-Exos-HIF1α combined with β-TCP scaffold material can significantly promote new bone formation and neovascularization in cranial critical-sized bone defects in rats’ with better results than BMSC-Exos combined with β-TCP scaffold. BMSC-Exos-HIF1α is expected to be an effective treatment for critical-sized bone defects in the clinic.

## Materials and Methods

This experiment was approved by the Ethics Committee of the Shanghai General Hospital, Shanghai Jiao Tong University School of Medicine. We have previously prepared mutant BMSC-HIF1α and related research papers have been published (Qi et al., 2013; Yang et al., 2014). The BMSC-HIF1α used in this experiment was provided by Professor Danping Liu’s team at Jinzhou Medical University.

### Isolation and Identification of BMSC-Exos-HIF1α

When BMSC-HIF1α reached 80–90% confluence, the medium was replaced with MGro-500 serum-free MSC medium (StemRD) and the cells were incubated for an additional 48 h. Conditioned medium from BMSC-HIF1α was collected and obtained by centrifugation at 3,00 × *g* for 10 min and 2,000 × *g* for 10 min, respectively. The supernatant was filtered in a Steritop^TM^ 0.22 μm filter sterilizer (Millipore, Billerica MA, United States), followed by ultrafiltration in a 15 mL centrifugal filter test tube (Millipore), and finally the supernatant was centrifuged at 4,000 × g to approximately 200 μL of liquid, the obtained suspension was washed twice with 15 mL PBS and ultrafiltered again with 4,000 × *g* to 200 μL. To purify the exosomes, the suspension was covered with 30% sucrose-D_2_O pads in sterile Ultra-Clear^TM^ tubes (Beckman Coulter, Brea, CA, United States) and ultracentrifuged at 1,00,000 × *g* for 2 h, the precipitated vesicles were thought to be exosomes. The precipitated exudate weight was suspended in 15 mL PBS and centrifuged at 4,000 × *g* in a centrifugal filtration test tube until the final volume was reduced to approximately 200 μL. All procedures were performed at 4°C. Tunable resistive pulse sensing analysis and western blotting for the identification of BMSC-Exos-HIF1α, data analysis using Izon Control Suite software v2.2 (Izon Science).

### Extraction and Culture of Rat Bone Marrow Mesenchymal Stem Cells

The experimental animals were 250–300 g Sprague-Dawley (SD) rats, Bone marrow from the humerus and femur of eight rats. Cells from rats were inoculated on a 10 cm^2^ plastic tissue culture plate containing 10% fetal bovine serum, 100 IU/mL penicillin, and 100 μg/mL streptomycin in MEM medium (MEM-α; Invitrogen, Carlsbad, CA, United States), incubated at 37°C in an incubator containing 5% CO2 for 48 h. Non-adherent cells were discarded and defined as first generation cells (P1) when they grew to 80% confluence, P3 cells were used for all experiments.

### Cell Proliferation Assay

The effect of BMSC-Exos-HIF1α on BMSCs proliferation was detected by Cell Count Kit-8 (CCK-8; Dojindo). Cells were randomly divided into three groups: control group (adding the same amount of cell medium); experimental control group (adding BMSC-Exos to 200 μg/mL concentration); experimental group (adding BMSC-Exos-HIF1α to 200 μg/mL concentration). 3 × 10^3^ BMSCs (p3) were grown in 96-well plates and enriched for 48 h. BMSC-Exos-HIF1α were then added to 96-well plates and incubated for an additional 1, 3, and 5 days, with the BMSC-Exos group performing the same operation. At the end of the incubation period, cells were incubated with 10 μL CCK-8 solution for approximately 4 h. Cell proliferation was calculated by measuring absorbance at 450 nm using an enzyme standardizer (BIO-TEK, ELX 800). The cell proliferation assay took three replicate wells for each group, and the experiment was repeated three times.

### Alkaline Phosphatase Activity Assay and Alizarin Red Staining

Three groups of cells were inoculated in 6-well plates and the destination cells were stimulated by three different methods, cultured in freshly formed osteogenic medium (OM) for 14 days, and ALP activity was determined using an ALP assay kit (Beyotime Institute of Biotechnology, Shanghai, China),the experiment was repeated three times with three replicate wells each group. Alizarin Red staining (Sigma, St Louis, MO, United States) was used to detect bone mineralization. The cells of each group were incubated with the corresponding experimental stimuli for 21 days, the supernatant was discarded, washed twice with PBS, fixed with 4% paraformaldehyde for 15 min, discarded, washed twice with PBS again, stained with 2% cisplatin red, stained at room temperature for 20 min, bone mineralization of specimens was observed by phase-contrast microscopy after the staining.

### Real-Time Quantitative Polymerase Chain Reaction

The effect of BMSC-Exos-HIF1α on the osteogenic differentiation of BMSCs was measured by Real-Time Quantitative Polymerase Chain Reaction (RT-qPCR) and all samples were tested in triplicate. Total RNA was extracted from three groups of cells by Trizol methods on day 3 and 7, respectively, RNA was reverse transcribed using the PrimeScript RT reagent kit, mRNA was reverse transcribed to cDNA and amplified by using the SYBR kit (TakaRa Bio, Shiga, Japan). β-actin as a control. The required primer information is listed in Table 1.

**TABLE 1:** Primer sequences used in RT-qPCR.

**Molecule**	**Primer sequences**
RUNX-2	CCGAGCTACGAAATGCCTCT
	GGACCGTCCACTGTCACTTT
ALP	GTTTTCTGTTCTGTAAGACGGG
	GCCGTTAATTGACGTTCCGA
COL1-A1	ACATGTTCAGCTTTGTGGACC
	AGGTTTCCACGTCTCACCAT

### Model of Cranial Deficiency in Rats

Thirty nine mature Sprague Dawley rats (12 weeks old, mean weight 250–300 g) were used for *in vivo* experimental studies. All surgical procedures are performed under general anesthesia and postoperative analgesic care with tramadol is ensured. All efforts are made to minimize the suffering of the animal. All operations are carried out under sterile conditions. We used classically porous β-TCP scaffolds with an average pore size of 500 μm and a classically porous β-TCP scaffold with 75% porosity (the subscaffold material has a diameter of 5 mm and a height of 2 mm) as BMSC-Exos and BMSC-Exos-HIF1α carriers. 200 μg BMSC-Exos and 200 μg BMSC-Exos-HIF1α drops were added to a sterile β-TCP holder under sterile conditions and freeze-dried for at least 4 h. Application of 4% hydrated chloral by intraperitoneal injection to anesthetize 30 rats, after successful anesthesia, the rats were placed prone on a fixed plate, head shaving, sterilized with iodine and spread sterile towels. A 1.5 cm sagittal incision is made in the scalp, the fascia is cut, the periosteum is peeled off the surface of the skull to both sides with a small bone peel, then a 5 mm diameter dental ring drill is used to drill a 5 mm diameter full-layer skull defect on each side of the skull surface on the median line (injury to the dura mater and brain tissue is strictly prohibited during surgery). The rats were randomly divided into three groups: β-TCP group (13): β-TCP stent only; 200 μg BMSC-Exos group + β-TCP (13): 200 μg BMSC-Exos + β-TCP stent; 200 μg BMSC-Exos-HIF1α group + β-TCP (13): 200 μg BMSC-Exos-HIF1α + β-TCP stent. Suture the surgical incision layer by layer after implanting the test material into the defect in each group. For three days after surgery, 40,000 units of gentamicin were injected daily to prevent infection. At 12 weeks postoperatively, the skulls of all experimental rats were removed after death by overanesthesia and stored in a 4% paraformaldehyde solution buffered in 0.1 M phosphate solution (pH 7.2).

### Micro-CT Analysis

Analysis of data related to bone defect areas using a Micro CT system (mCT-80, Scanco Medical, Brüttisellen, Switzerland). The fixed specimens were subjected to Micro-CT (SKYSCAN 1176, Bruker) with the following parameters: operating voltage 80 KV, exposure time 240 ms, thickness of the specimen scanned 18 μm. Skyscan software is included in the micro-CT system used in this experiment and Skyscan software includes CT-An, CT-Vol, CT-Vox and other applications. The 2D images were obtained directly after Micro-CT scan, the 3D reconstruction images were constructed using CT-Vol application, and the bone mineral density (BMD) and bone volume fraction (BV/TV) were automatically determined by using CT-An application. BMD and BV/TV values in the bone defect area were assessed for new bone formation by using auxiliary software (Scanco), each group randomly selected 6 samples for analysis and detection.

### Sequential Fluorescent Labeling

Intraperitoneal injections of tetracycline hydrochloride (TE, 25 mg/kg bw), alizarin red (AL, 30 mg/kg bw) and calcitonin (CA, 20 mg/kg bw) were given to rats at 2, 4, and 6 weeks postoperatively, respectively. Application of confocal laser scanning microscopy (CLSM, Leica) to examine sections for data analysis and image acquisition by three different fluorescence reactions. Yellow fluorescence images indicate TE, red fluorescence images indicate AL, and green fluorescence images indicate CA, indicating new bone formation and new bone tissue mineralization at weeks 2, 4, and 6 after surgery, respectively. Percentages of three staining results for TE, AL and CA were analyzed by histomorphometric analysis, each group randomly selected six samples for analysis and detection.

### Microfil Perfusion Testing

Rats killed by chloral hydrate overanesthesia were perfused with Microfil infusion (Flowtech, Carver, MA, United States). A longitudinal incision was made in the rat’s chest to open the descending aorta, a vascular catheter was placed in the left ventricle, the inferior vena cava was cut, 20 mL of heparinized saline was repeatedly perfused, then 20 mL of Microfil was perfused at 2 mL/min, and the vessel was ligated with sutures after perfusion, and the rat was kept in a 4°C refrigerator overnight. The cranial specimens were kept in formalin solution for more than 24 h, decalcified in decalcification solution for about 2 weeks, and then the specimens were scanned under the Micro CT system and the neovascularization in the cranial defect was represented by 3D reconstruction image. The area and number of local blood vessels in bone defects were evaluated by CT-Vox program, each group randomly selected 6 samples for analysis and detection.

### Histomorphology and Immunohistochemistry Analysis

Six skull samples were randomly selected from each group and dehydrated through a concentration gradient from 70 to 100% ethanol and then embedded in polymethyl methacrylate (PMMA). The cranial specimens were then cut into longitudinal sections 150–200 μm thick using a hard tissue biopsy machine (Leica Microsystems, Wetzlar, Germany), glued onto plastic carrier slides and sandpapered thin and polished to a final thickness of about 50 μm. Sections were fluorescently labeled with laser confocal scanning microscopy (Leica) to quantify new bone formation and bone mineralization at four locations, and the average of four measurements was calculated to assess the mean values for each group. Sections were stained with van Gieson’s to evaluate new bone formation, with red areas representing new bone formation and black areas representing β-TCP stents. Three sections were randomly selected from each skull samples, with 18 slices in each group, a total of 54 slices were used for analysis of new bone formation. Image acquisition for quantitative evaluation of the area of new bone formation by Image J software. Another portion of each cranial specimen was decalcified in a decalcification solution for approximately two weeks, dehydrated by gradient ethanol, encased in paraffin, cut into 5 μm thick sections, and immunohistochemically analyzed for osteocalcin (OCN) and CD31 to assess bone formation and angiogenesis, respectively.

### Statistical Analysis

Statistical analysis was performed using SPSS 22.0 software (Release 22.0; SPSS, Chicago, IL, United States). All of the data were shown as the mean ± standard deviation (SD). The Kolmogorov-Smirnov test was used to analyze whether the data obeyed a normal distribution. The test of homogeneity of variance was used to analyze whether the data met the homogeneity of variance. For the data with normal distribution, the difference between groups was calculated by one-way analysis of variance (ANOVA), otherwise, non-parametric test was used. The difference was significant when *P*-value < 0.05.

## Results

### Identification of BMSC-Exos-HIF1α

**FIGURE 1:**
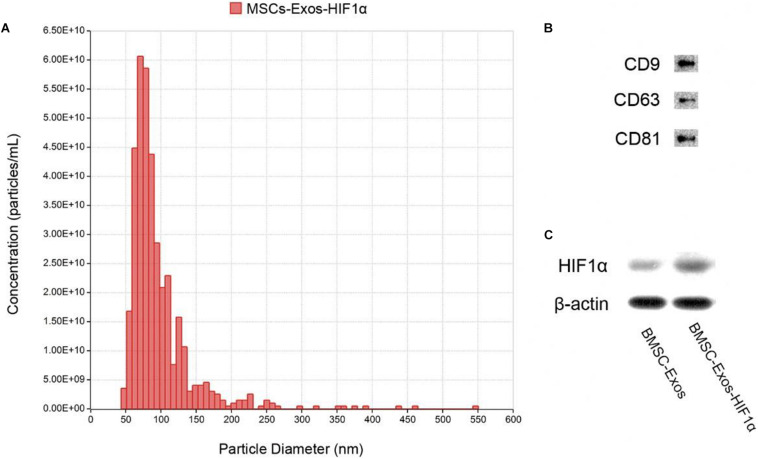
Identification of BMSC-Exos-HIF1α. **(A)** Distribution of particle size and concentration of BMSC-Exos-HIF1α detected by TRPS. **(B)** Expression of surface proteins CD9, CD63, and CD81 in BMSC-Exos-HIF1α as determined by Western blotting. **(C)** HIF1α expression of BMSC-Exos-HIF1α as determined by Western blotting.

Identification of BMSC-Exos-HIF1α using Tunable Resistive Pulse Sensing (TRPS) analysis and Western blotting. The results showed that most of the particles in the sample were distributed in the range of 50–150 nm (Figure 1A), and the experimental results showed that they belonged to the BMSC-Exos-HIF1α. Western blotting results showed that these particles express characteristic surface markers (CD9, CD63, and CD81) (Figure 1B) and HIF-1α (Figure 1C), which further confirmed that they are BMSC-Exos-HIF1α.

### BMSC-Exos-HIF1α Promotes Proliferation and Osteogenic Differentiation of BMSCs

**FIGURE 2:**
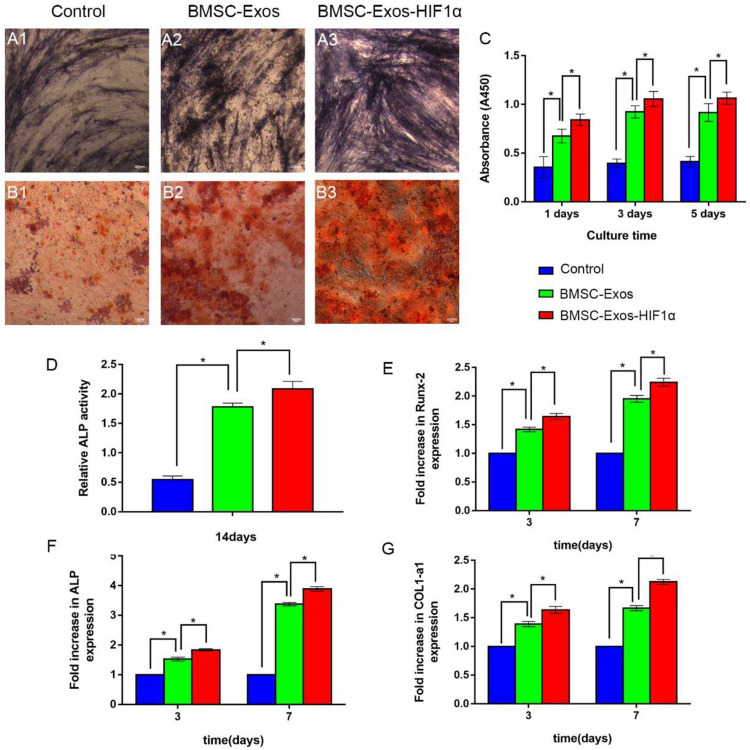
BMSC-Exos-HIF1α promotes proliferation and osteogenic differentiation in bone marrow mesenchymal stem cells. Alkaline phosphatase activity assay (14 days) **(A1–A3)** and quantitative analysis **(D)**, Alizarin red staining (21 days) **(B1–B3)** to determine the effect of BMSC-Exos-HIF1α on osteogenic differentiation of BMSCs. **(C)** CCK-8 method to assess the effect of BMSC-Exos-HIF1α on the proliferation of BMSCs. RT-qPCR method to detect gene expression levels of RUNX-2 **(E)**, ALP **(F)**, and COL1 **(G)** on the third and seventh day of culture. All tests were repeated three times (* indicates significant difference, *P* < 0.05; scale bar: 100 μm).

The CCK-8 method was used to evaluate the effect of BMSC-Exos-HIF1α on BMSCs proliferation (Figure 2C). The experimental results showed a statistically significant difference between the 200 μg/mL BMSC-Exos-HIF1α group and the control, 200 μg/mL BMSC-Exos group (*P* < 0.05). The results showed that BMSC-Exos-HIF1α was effective in promoting the proliferation of BMSCs and was more effective than BMSC-Exos. ALP activity as an early marker of osteogenic differentiation, the results of this experiment were shown in Figures 2A1–A3, more pronounced ALP staining was observed in cells treated with 200 μg/mL BMSC-Exos-HIF1α. Quantitative analysis of ALP activity (Figure 2D) showed a statistically significant difference (*P* < 0.05) between the 200 μg/mL BMSC-Exos-HIF1α group and the control, 200 μg/mL BMSC-Exos group at day 14 of culture. Alizarin red staining showed significantly enhanced mineral deposition in the 200 μg/mL BMSC-Exos-HIF1α group compared to the control and 200 μg/mL BMSC-Exos group (Figures 2B1–B3). Both ALP activity and alizarin red staining showed that BMSC-Exos-HIF1α can effectively promote osteogenic differentiation of BMSCs, and the effect is better than BMSC-Exos. RT-qPCR assay showed that mRNA expression of RUNX-2, ALP and COL1 was significantly increased in cells treated with BMSC-Exos-HIF1α, and there was a statistically significant difference between the 200 μg/mL BMSC-Exos-HIF1α group and the control, 200 μg/mL BMSC-Exos group (*P* < 0.05). RT-qPCR results showed that BMSC-Exos-HIF1α can up-regulate the expression of osteogenesis-related genes in BMSCs with better effect than BMSC-Exos (Figures 2E–G). In summary, *in vitro* studies show that BMSC-Exos-HIF1α can effectively stimulate the proliferation of BMSCs and enhance their osteogenic differentiation, which is superior to BMSC-Exos.

### Micro-CT Analysis of New Osteogenesis in the Cranial Defect Area

**FIGURE 3:**
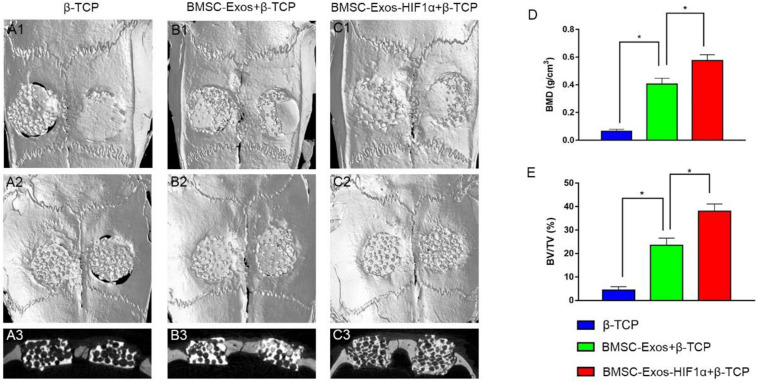
Micro-CT analysis of BMSC-Exos-HIF1α + β-TCP promoting new bone regeneration. Analysis of three-dimensional Micro-CT images at 12 weeks post-modeling, external cranial reconstruction **(A1–C1)**, internal cranial reconstruction **(A2–C2)**, sagittal position **(A3–C3)**; Quantitative analysis of bone mineral density (BMD) **(D)** and bone volume fraction (BV/TV) **(E)** at 12 weeks after surgery (*n* = 6,* indicates statistically significant difference, *P* < 0.05).

**FIGURE 4:**
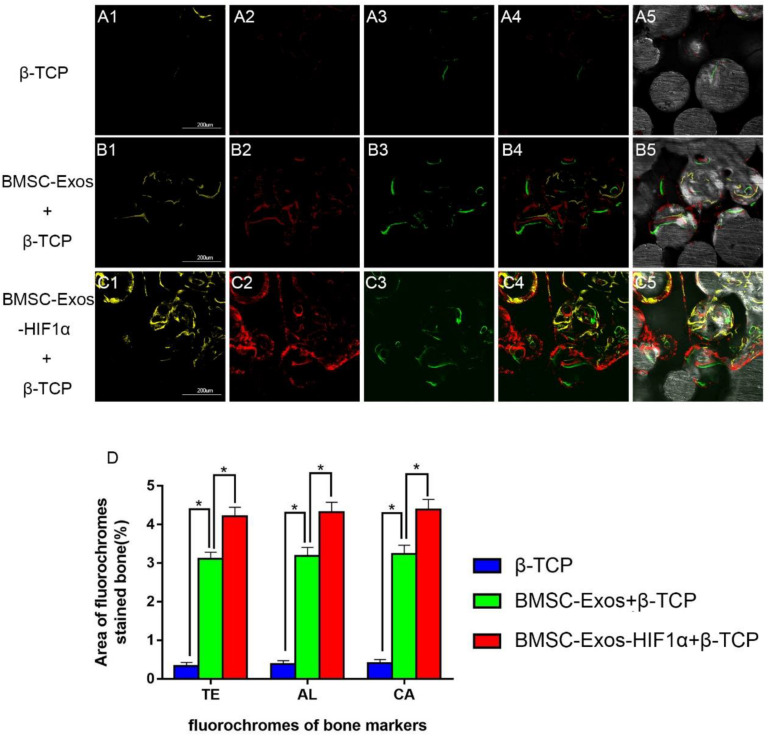
Sequential fluorescent labeling analysis of BMSC-Exos-HIF1α + β-TCP to promote new bone regeneration. Yellow images **(A1–C1)** indicate TE, red images **(A2–C2)** indicate AL, and green images **(A3–C3)** indicate **CA**, indicating new bone formation and mineralization at postoperative weeks 2, 4, and 6, respectively. Combined images of three fluorescent staining results **(A4–C4)**, combined images of three fluorescent staining results with normal images **(A5–C5)**, histomorphometric quantitative analysis of TE, AL, and CA fluorescence expression at 12 weeks after surgery **(D)**. (*n* = 6,* indicates statistically significant difference, *P* < 0.05; scale bar: 200 μm).

Micro-CT 3D reconstruction image analysis showing morphology of newly formed bone (Figures 3A1–C1, 3A2–C2). In the sagittal view (Figures 3A3–C3), more newly formed bone can be observed in the 200 μg BMSC-Exos-HIF1α + β-TCP group than in the 200 μgBMSC-Exos + β-TCP group or β-TCP group. Quantitative analysis showed that the BMD of the bone defect area was significantly higher in the 200 μgBMSC-Exos-HIF1α + β-TCP group (0.575 ± 0.043 g/cm^3^) than in the 200 μgBMSC-Exos + β-TCP group (0.406 ± 0.041 g/cm^3^) or β-TCP group (0.064 ± 0.016 g/cm^3^) (*P* < 0.05) (Figure 3D). BV/TV in the bone defect area was significantly higher in the 200 μgBMSC-Exos-HIF1α + β-TCP group (38.03 ± 3.08%) than in the 200 μgBMSC-Exos + β-TCP group (23.51 ± 3.04%) or β-TCP group (4.46 ± 1.42%) (*P* < 0.05) (Figure 4E). The above results indicate that BMSC-Exos-HIF1α + β-TCP can effectively promote new bone regeneration, and the effect is better than BMSC-Exos + β-TCP.

### Fluorochrome Labeling Histomorphometric Analysis

Figure 4 shows the results of new bone formation and mineralization by fluorescence labeling analysis at weeks 2, 4, and 6 post-modeling. At week 2, the percentage of TE markers (yellow) in the 200 μg BMSC-Exos-HIF1α + β-TCP group (4.21 ± 0.23%) was significantly higher than in the 200 μg BMSC-Exos + β-TCP group (3.11 ± 0.17%) or β-TCP group (0.34 ± 0.09%) (*P* < 0.05) (Figures 4A1–C1). At week 4, the percentage of AL markers (red) in the 200 μg BMSC-Exos-HIF1α + β-TCP group (4.32 ± 0.26%) was significantly higher than in the 200 μg BMSC-Exos + β-TCP group (3.18 ± 0.22%) or the β-TCP group (0.38 ± 0.08%) (*P* < 0.05) (Figures 4A2–C2). At week 6, the percentage of CA markers (green) in the 200 μgBMSC-Exos-HIF1α + β-TCP group (4.39 ± 0.27%) was significantly higher than in the 200 μg BMSC-Exos + β-TCP group (3.24 ± 0.23%) or β-TCP group (0.41 ± 0.09%) (*P* < 0.05) (Figures 4A3–C3). The above results indicate that BMSC-Exos-HIF1α + β-TCP can effectively promote the regeneration of new bone in the bone defect area and its effect is better than that of BMSC-Exos + β-TCP.

### Analysis of Neovascular Formation in Bone Defects

**FIGURE 5:**
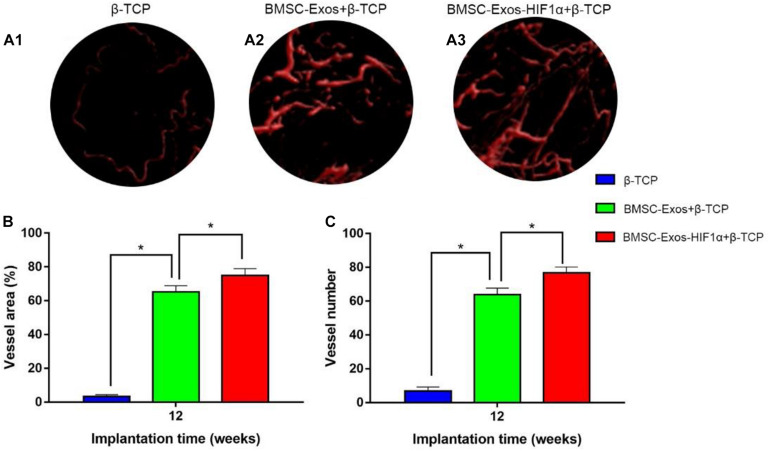
Results of BMSC-Exos-HIF1α + β-TCP promoted angiogenic by Microfil perfusion. **(A1–A3)** Three-dimensional reconstructive images showing neovascular formation in the area of bone defects. **(B)** Analysis of the area of neovascularization in the bone defect area, **(C)** Analysis of the number of neovascularization in the bone defect area. (*n* = 6,* indicates statistically significant difference, *P* < 0.05).

Micro-CT image analysis showed that the 200 μgBMSC-Exos-HIF1α + β-TCP group had more neovascularization compared to the 200 μgBMSC-Exos + β-TCP group, β-TCP group (Figures 5A1–A3). Quantitative analysis of the area of neovascular formation (Figure 5B) and the number of neovessels (Figure 5C) showed the same results. There was a statistically significant difference in the number of neonatal vessels in the 200 μgBMSC-Exos-HIF1α + β-TCP group (76.8 ± 3.4) compared to the 200 μgBMSC-Exos + β-TCP group (63.9 ± 3.8) and the β-TCP group (6.8 ± 2.4) (*P* < 0.05). The 200 μgBMSC-Exos-HIF1α + β-TCP group had the largest area of neovascularization (74.91 ± 4.05%) and a statistically significant difference compared to the 200 μgBMSC-Exos + β-TCP group (65.24 ± 3.65%) and the β-TCP group (3.38 ± 0.98%) (*P* < 0.05). The experimental results showed that BMSC-Exos-HIF1α + β-TCP can effectively promote neovascularization in the bone defect area, and the effect is better than BMSC-Exos + β-TCP.

### Histological and Immunohistochemical Analysis

**FIGURE 6:**
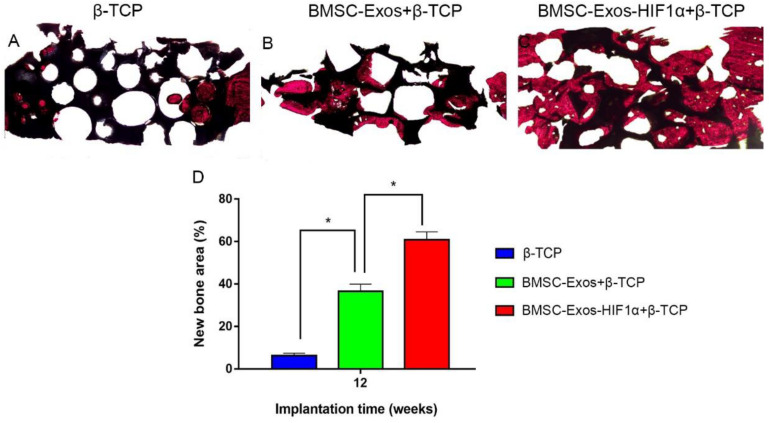
Histological analysis of BMSC-Exos-HIF1α + β-TCP scaffold promoting new bone regeneration. **(A–C)** Results of VG staining of hard tissue sections, red represents the area of new bone formation and black represents the part of the remaining scaffold material, **(D)** Results of quantitative histomorphological analysis 12 weeks after surgery (X40). (*n* = 6,* indicates statistically significant difference, *P* < 0.05).

Analysis of the Van Gieson staining results of undecalcified samples showed a significant increase in new osteogenesis in the 200 μg BMSC-Exos-HIF1α + β-TCP group (60.87 ± 3.71%), the area of new bone formation was significantly larger than 200 μg BMSC-Exos + β-TCP group (36.52 ± 3.47%) and β-TCP group (6.26 ± 1.19%) (*P* < 0.05), and there was a statistically significant difference between the three groups (*P* < 0.05) (Figure 6). The histological staining results showed the same trend as the results of the new bone regeneration Micro-CT analysis.

**FIGURE 7:**
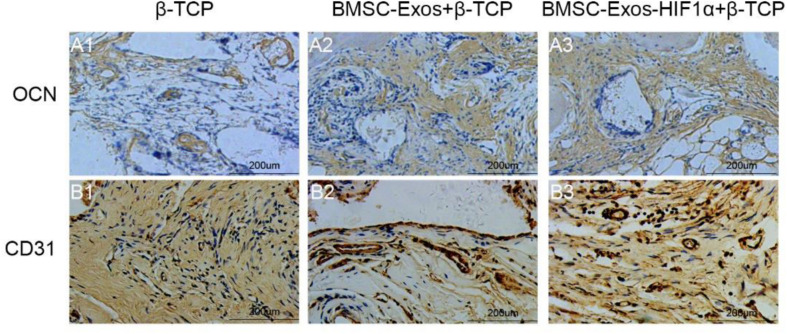
Immunohistochemical analysis of BMSC-Exos-HIF1α + β-TCP for promoting new bone regeneration and neovascularization. Application of immunohistochemical analysis to detect new osteogenesis and neovascularization, OCN **(A1–A3)** and CD31 **(B1–B3)** in samples. (scale bar: 200 μm).

Immunohistochemical staining of decalcified skulls for the osteogenic marker OCN and the neovascularization marker CD31 (Figure 7), OCN results showed almost no significant positive staining in the β-TCP group (Figure 7A1), however, OCN-positive staining was evident in the 200 μgBMSC-Exos + β-TCP group (Figure 7A2) and the 200 μgBMSC-Exos-HIF1α + β-TCP group (Figure 7A3). 200 μgBMSC-Exos-HIF1α + β-TCP showed the most obvious positive staining results for OCN. Compared to the β-TCP group (Figure 7B1), the CD31 positive staining in the 200 μg BMSC-Exos-HIF1α + β-TCP group (Figure 7B2) or the 200 μg BMSC-Exos-HIF1α + β-TCP group (Figure 7B3) was significant, the most pronounced CD31 positive staining was observed in the 200 μg BMSC-Exos-HIF1α + β-TCP group. The results showed that BMSC-Exos-HIF1α + β-TCP can effectively promote the expression of osteogenic markers and vascular neovascularization markers in the bone defect area, and the effect was better than that of BMSC-Exos + β-TCP.

## Discussion

Due to the limited inherent regenerative capacity of bone, critical-sized bone defects caused by trauma, bone tumor removal, etc. are difficult to cure through autologous bone regeneration. How to repair critical-sized bone defects has always been a difficult task in orthopedics. Although autologous bone grafting is considered the gold standard for repairing bone defects, its application is limited by donor site morbidity, limited availability, and unpredictable autologous resorption, among other limitations (Rogers and Greene, 2012). Advances in bone tissue engineering provide a new avenue for bone defect repair, and tissue engineering strategies that use specific structures to repair bone defects have advantages over current bone grafting techniques (McGovern et al., 2018). Our results show for the first time that BMSC-Exos-HIF1α can effectively stimulate the proliferation and osteogenic differentiation of BMSCs and is more effective than BMSC-Exos, BMSC-Exos-HIF1α + β-TCP effectively promotes new bone regeneration and neovascularization in a rat cranial defect model, superior to BMSC-Exos + β-TCP.

In bone defect repair, angiogenesis and osteogenesis are closely related, with HIF-1α playing an important role. HIF-1α found to promote differentiation of BMSCs toward osteogenic cells (Ding et al., 2013; Zhang J. et al., 2018); H1F-1α and Runx2 interact via the Runt domain to upregulate VEGF expression to promote angiogenesis, and to upregulate Runx2 expression (Kwon et al., 2011; Lee et al., 2012). Runx2 is a transcription factor that plays an important regulatory role in bone formation and development and is essential for osteogenic cell differentiation, Runx2 as a target gene of the Wnt/β-catenin signaling pathway activates the Wnt/β-catenin signaling pathway to promote new bone formation (Lin et al., 2011; Cai et al., 2016; Thiagarajan et al., 2017; Vega et al., 2017). In addition, HIF-1α was found to significantly upregulate CXCL12 expression at the ischemic site, prompting CXCL12 to diffuse to the periphery, forming a concentration gradient, ultimate promotion of mobilization and homing of EPCs via CXCL12/CXCR4 bio-axis enhances angiogenesis in the ischemic zone (Bianco et al., 2015; Kawakami et al., 2015). However, the physiologically expressed HIF-1α is degraded by the body within minutes under normoxic conditions. It was found that the mechanism by which HIF-1α is rapidly degraded by the organism under normoxic conditions is the HIF-1α subunit functional region (CDS region) encoding 826 amino acids, which has an oxygen-dependent degradation structural domain (ODD) consisting of more than 200 amino acids, Hydroxylation of the 402nd and 564th proline residues in the ODD of the HIF-1α molecule catalyzed by proline hydroxylase (oxygen is required for this hydroxylation process), and the hydroxylated HIF-1α is rapidly degraded by ubiquitin protease (Jin et al., 2009; Kim et al., 2009; Wei et al., 2010). In contrast, under hypoxic conditions, because proline 402, 564 cannot be hydroxylated, HIF-1α accumulates and is transferred to the nucleus, where it activates the hypoxia-responsive gene cluster. It was also shown that the transcriptionally active region that determines HIF-1α is located at position 803 asparagine at its carboxyl terminus (CAD) and that blocking its hydroxylation with chemical inhibitors or gene recombination leads to strong transcriptional activity of HIF-1α. Thus, the important structural regions of HIF-1α include not only proline 402 and 564 in the ODD region, but also asparagine 803 in the CAD region. In this subject, 3-point mutant HIF-1α was successfully prepared and BMSCs modified with 3-point mutant HIF-1α were found to be effective in promoting vascularization in bone ischemic areas *in vivo* (Qi et al., 2013; Yang et al., 2014).

Bone marrow mesenchymal stem cells have superior osteogenic potential (Reinisch et al., 2015), can differentiate into osteoblasts and promote vascular regeneration through paracrine action (Phinney and Prockop, 2007; Satija et al., 2009; Wei et al., 2014), but the direct application of stem cells is limited by factors such as immune rejection, chromosomal variation, etc., (Wang et al., 2012). There are many components in exosomes (Phinney and Pittenger, 2017; Zhang S. et al., 2018), exosomes act as important participants in intercellular signaling by transferring functional proteins, RNA, microRNA and lncRNA between cells and can significantly reduce immune response (Poe and Knowlton, 2018; Zhao et al., 2020). The biological function of stem cells is mainly achieved by stimulating effector cells with specific biologically active molecules carried in the exocrine body. Studies have shown that BMSC-Exos can promote bone regeneration by promoting osteogenesis and angiogenesis (Nakamura et al., 2015; Xue et al., 2018; Takeuchi et al., 2019; Lee et al., 2020; Xu et al., 2020; Zhang L. et al., 2020). Therefore, our study aimed to investigate the effect of BMSC-Exos-HIF1α on the proliferation and osteogenic differentiation of BMSCs, investigating the biological function of BMSC-Exos-HIF1α loading on β-TCP scaffold material to repair bone defects in rats. However, our study also had some limitations that we did not explore the signal pathways and molecular mechanisms of BMSC-Exos-HIF1α in repairing bone defects through promoting osteogenesis and angiogenesis, which will be further explored in our follow-up studies.

Assessment of the biological function of BMSC-Exos-HIF1α to repair bone defects using a rat skull defect model. Micro-CT quantitative analysis showed that the application of BMSC-Exos-HIF1α + β-TCP scaffold significantly increased the formation of new bone in the skull defect area of rats, and the amount of new bone formation was more than that of BMSC-Exos + β-TCP. Histological analysis showed that the β-TCP group showed almost no new bone formation in the area of bone defects, while both the BMSC-Exos + β-TCP group and the BMSC-Exos-HIF1α + β-TCP group showed the greatest amount of new bone formation, Histological analysis results are consistent with Micro-CT quantitative analysis. Microfil perfusion is used to evaluate the neovascularization of bone defect area in this experiment, analysis of both the area of neovascularization and the number of neovessels showed more neovascularization in both the BMSC-Exos + β-TCP group and the BMSC-Exos-HIF1α + β-TCP group, the BMSC-Exos-HIF1α + β-TCP group showed the most neovascular formation. OCN and CD31 are common markers in studies of osteogenic differentiation and neovascularization, respectively. Analysis of immunohistochemical results showed that OCN and CD31 had only a small amount of positive staining expression in the bone defect area in the β-TCP group, and more positive staining results in the BMSC-Exos-HIF1α + β-TCP group than in the BMSC-Exos + β-TCP group.

In summary, BMSC-Exos-HIF1α + β-TCP could significantly repair critical-sized bone defects by enhancing neogenesis and neovascular formation *in vivo*.

## Conclusion

In our current study, we demonstrate for the first time that BMSC-Exos-HIF1α effectively stimulates the proliferation and osteogenic differentiation of BMSCs, and is more effective than BMSC-Exos. BMSC-Exos-HIF1α combined with β-TCP scaffolds is superior to BMSC-Exos combined with β-TCP scaffolds in repairing critical-sized bone defects by promoting new bone regeneration and neovascularization in the defect area.

## Data Availability Statement

All datasets presented in this study are included in the article/supplementary material.

## Ethics Statement

The animal study was reviewed and approved by The Ethics Committee of the Shanghai General Hospital, Shanghai Jiao Tong University School of Medicine.

## Author Contributions

XQ and DH designed the tests. RW and ZW carried out the tests. CY analyzed experimental results. JT directed the experiments. CY and RW wrote this manuscript, two authors contributed equally to this work. Authors mentioned before modified this manuscript. WY, JZ and CQY provided valuable comments on the revised manuscript after submission.

## Conflict of Interest

The authors declare that the research was conducted in the absence of any commercial or financial relationships that could be construed as a potential conflict of interest.
